# Complimentary Imaging Modalities for Investigating Obscure Gastrointestinal Bleeding: Capsule Endoscopy, Double-Balloon Enteroscopy, and Computed Tomographic Enterography

**DOI:** 10.1155/2016/8367519

**Published:** 2015-12-27

**Authors:** Ye Chu, Sheng Wu, Yuting Qian, Qi Wang, Juanjuan Li, Yanping Tang, Tingting Bai, Lifu Wang

**Affiliations:** Department of Gastroenterology, Ruijin Hospital Affiliated to Shanghai Jiao Tong University School of Medicine, Shanghai 200025, China

## Abstract

*Objectives*. The complimentary value of computed tomographic enterography (CTE) and double-balloon enteroscopy (DBE) combined with capsule endoscopy (CE) was evaluated in the diagnosis of obscure gastrointestinal bleeding (OGIB). *Methods*. Patients who received CE examinations at Ruijin Hospital between July 2007 and July 2014 with the indication of OGIB were identified, and those who also underwent DBE and/or CTE were included. Their clinical information was retrieved, and results from each test were compared with findings from the other two examinations. *Results*. The overall diagnostic yield of CE was comparable with DBE (73.9% versus 60.9%) but was significantly higher than the yield of CTE (87% versus 25%, *p* < 0.001). The diagnostic yield of angiodysplasia at CE was significantly higher than CTE (73% versus 8%, *p* < 0.001) and DBE (39.1% versus 17.4%, *p* = 0.013), while no significant difference was found between the three approaches for small bowel tumors. DBE and CTE identified small bowel diseases undetected or undetermined by CE. Conversely, CE improved diagnosis in the cases with negative CTE and DBE, and findings at initial CE directed further diagnosis made by DBE. *Conclusions*. Combination of the three diagnostic platforms provides complementary value in the diagnosis of OGIB.

## 1. Introduction

Upper and lower gastrointestinal (GI) endoscopies are used in most cases to determine the location of gastrointestinal bleeding, a common symptom of disease in the GI tract. However, the source of nearly 5% of GI bleeding remains undetected, after completion of both upper and lower endoscopy procedures [1]. This is referred to as obscure GI bleeding (OGIB). The majority of lesions of OGIB are eventually located in the small intestine, which is beyond reach of conventional endoscopy. In the recent ten years, the application of video capsule endoscopy, computed tomographic enterography, and double-balloon enteroscopy have revolutionized intestinal imaging and greatly improved diagnosis of small bowel diseases. This retrospective study was designed to compare the complimentary value of CTE and DBE combined with CE in the diagnosis of OGIB.

## 2. Patients and Methods

### 2.1. Study Cohorts

Approval to carry out this study was granted by the ethics committee of Ruijin Hospital affiliated to Shanghai Jiao Tong University. Patients who underwent capsule endoscopy at Ruijin Hospital (between July 2007 and July 2014) with the indication of OGIB were identified, and those who also underwent DBE and/or CTE before or after CE were included for this study. A total of 121 patients met the inclusion criteria. CE was performed in all patients; CTE and DBE were performed in 100 (82.6%) and 46 (38.0%) of the patients, respectively. The interval between CE and DBE examinations was within 1 week, and the interval between CE and CTE was within 1 month. Antegrade and retrograde DBE were not combined in all patients. Medical records were retrieved and clinical information was extracted for all selected patients.

### 2.2. Capsule Endoscopy Technique

All CE examinations were performed using the OMOM capsule endoscopic device (Jinshan Science and Technology Group Co., Ltd, Chongqing, China). Patients were instructed to take a light diet free of fruit and vegetables the day before examination and ingest 2 L polyethylene glycol-based electrolyte solution 12 hours prior to the test, followed by an overnight fast for bowel preparation. Patients received 300 mg simethicone 60 minutes before the test. Capsules were swallowed on the morning of the test and allowed to run for at least 8 hours. The accumulated images were transferred to a computer workstation and reviewed by experienced gastroenterologists. CE results were regarded as “positive” if they could explain the cause of clinical presentation and other lesions accountable for the disease could not be determined.

### 2.3. Double-Balloon Enteroscopy Technique

The DBE system consists of a video endoscope (Fujinon EN-450P5/20; Fujinon Inc., Saitama, Japan) with a working length of 200 cm and an outer diameter of 8.5 mm, together with an overtube of 145 cm in length and 12.2 mm in diameter. Two latex balloons are attached to the tips of the endoscope and the overtube. Patients scheduled for the oral procedure had overnight fast, and those scheduled for the anal procedure received bowel preparation used for CE procedure the day before the examination. Biopsy specimens were obtained for pathology during DBE when necessary. Of all cases in our study, most DBE (38 out of 46 cases) were performed after CE screening, and the initial route of DBE was chosen according to the predicted location of bleeding lesion based on CE findings and the patient's medical history. In the remaining cases where DBE was done prior to CE, route was chosen based on patient's medical history.

### 2.4. Dual-Phase CT Enterography Technique

The dual-phase CTE scanning was performed on the multislice multidetector CT scanner (GE Medical Systems, Milwaukee, WI, USA) using the following parameters: 120 kVp, 280–320 mA, and a pitch of 1.0. Following bowel preparation used for CE procedure on the previous day, patients were given 2000 mL 2.5% iso-osmotic mannitol solution to drink approximately 45 min prior to the examination, in four 500 mL doses at an interval of 10 min, to achieve optimal small bowel distension. Scanning was performed from the porta hepatis to pubic symphysis, and images were obtained at 20–25 s and 60–65 s for dual-phase examinations after intravenous injection of the nonionic contrasts (Ioversol, Optiray 320; Tyco Healthcare, Montreal, Canada) for a total of 90 to 120 mL (1.5 mL/kg of body weight) via the antecubital vein at the rate of 3 mL/s. Images were reconstructed with 1.25 mm section width and 1.25 mm reconstruction interval using the workstation (ADW4.2 and ADW4.4). Of all the CTE procedures done in our study, most CTE (77 out of 100 cases) were performed prior to CE screening, and the remaining was done after CE.

### 2.5. Statistical Analysis

Data were analyzed with SPSS 17.0 (SPSS Inc., Chicago, Illinois, USA). A diagnostic yield was calculated by dividing the total number of patients who underwent the specific test by the number of cases with positive findings. Sensitivities were calculated from 2 × 2 contingency tables, and 95% confidence intervals were computed using the exact binomial method. Matched pair comparisons of positive findings on different tests were analyzed by the McNemar test. *p* < 0.05 was considered significant.

## 3. Results

### 3.1. Study Population

Among the 121 patients included in the study, 60 (49.6%) were males. The average age was 51.1 years. Of all the patients, one had capsule retention and was later diagnosed with small bowel carcinoma. Based on the results of combined imaging approaches performed on each patient, positive diagnosis was made in all of them, including angiodysplasia (83, 68.6%), small bowel tumors (SBT, 27, 22.3%), diverticula (6, 5.0%), and Crohn's disease (CD, 5, 4.1%). SBT and diverticula were diagnosed after surgical or pathological confirmation. Angiodysplasia and CD were diagnosed after our follow-up showed response to corresponding treatment in those patients. The characteristics and findings of the study population are shown in Table 1.

### 3.2. Findings on CE

Positive findings were observed on CE in 115 (95%) of the patients included in the study, and 104 cases (86%) were confirmed. The most common finding was angiodysplasia (86, 71.1%). Among those diagnosed with angiodysplasia, 5 cases were misdiagnosed by CE; tumors in 4 cases and Meckel's diverticulum in 1 case were determined with CTE and/or DBE after CE was performed and regarded as the main lesion of disease. The second common finding was small bowel masses (15, 12.4%), and the biopsy specimens obtained through follow-up DBE or surgery were verified to be tumors pathologically. Active bleeding with undetermined focus of lesion was observed in 6 cases, which was later found to be SBT in 4 cases and angiodysplasia in 2 cases by the other two methods. CD was diagnosed in 5 cases. Three Meckel's diverticula were detected and later confirmed. In the remaining 6 cases, findings were negative or nonspecific, but CTE and/or DBE were able to pick up SBT or diverticula in those patients. Of all patients undergoing CE examinations, capsules did not reach the cecum for complete small bowel examination in 30 cases (24.8%). However, positive findings were made in all the cases. Among the 30 cases, 5 were diagnosed with SBT, 5 were CD, and 2 were confirmed as diverticula, which might account for the high incompletion rates because of potential intestinal stricture caused by the diseases.

### 3.3. Findings on CTE

Positive findings were observed in 27 (27%) of the patients undergoing CTE, and 25 cases (25%) were confirmed. Suspected SBT were found in 17 (17%) cases, 15 of which were confirmed pathologically, and the remaining two were not confirmed after follow-up with the patients for two years. Focal intramural vessel dilatation with active bleeding was observed in 8 (8%) patients; all were later diagnosed with angiodysplasia by CE, with fresh blood also detected on CE in 3 of the cases. CD was observed in one case. Diverticulum was found in one patient and later confirmed by surgery. In the remaining 73 (73%) cases where nonspecific or negative findings were given, the majority (64, 64%) were diagnosed with angiodysplasia by CE. CTE failed to detect SBT in 6 patients and provide evidence of CD in 3 patients.

### 3.4. Findings on DBE

Positive findings were observed in 29 (63%) of the patients undergoing DBE, and 28 cases (60.9%) were confirmed. SBT were identified in 15 (32.6%) cases, which were confirmed pathologically. Meckel's diverticula were observed in 5 (10.9%) patients, later confirmed in surgery. Angiodysplasia was found in 9 (19.6%) cases, but tumor was identified by CTE in one of them with confirmation from surgery. DBE was negative in the remaining 17 (37.0%) cases. Among them, 12 cases of angiodysplasia, 2 cases of SBT (confirmed pathologically), 2 cases of CD, and one case of diverticulum were detected by CE and/or CTE. In the cases where DBE was negative, the lesions found by CE were theoretically supposed to be within reach of DBE via the chosen route.

### 3.5. Comparison of Findings on CE, CTE, and DBE

Overall, paired comparisons demonstrated a better diagnostic yield of CE compared with CTE (87% versus 25%, *p* < 0.001), but there was no significant difference between CE and DBE (73.9% versus 60.9%). Statistical analysis of subgroups showed higher yield of angiodysplasia at CE compared with CTE (73% versus 8%, *p* < 0.001) and DBE (39.1% versus 17.4%, *p* = 0.013), while no significant difference was found between the three approaches for SBT. Other lesions were not compared statistically due to limited sample size.

Next, we compared patients who had both CE and DBE, and the results showed higher miss rates of CE in the diagnosis of SBT and diverticulum versus DBE (38.9% versus 16.7% and 40% versus 0%, resp.), while DBE missed more angiodysplasia cases compared with CE (60% versus 10%) (Table 2). Among patients who had both CE and CTE, the miss rate of CE was higher in the diagnosis of SBT but much lower in the diagnosis of angiodysplasia as compared with CTE (52.4% versus 33.3% and 1.4% versus 89.2%) (Table 3). For patients who had all three procedures, CTE and DBE showed much higher miss rates in the diagnosis of angiodysplasia compared with CE (90.9% and 81.8% versus 9.1%), and the miss rates of CE and CTE were higher in the diagnosis of SBT compared with DBE (50% and 50% versus 25%) (Table 4).

We also evaluated the effect of CE and DBE sequence on the diagnostic outcome. In the 8 cases where DBE was performed prior to CE, DBE was negative in all, while CE detected small bowel lesions. In the 37 cases where DBE was performed after CE and with the guidance of previous CE results, DBE confirmed CE findings in 19 cases and found lesions undetermined or undetected by CE in 9 cases. In the remaining one case where DBE was performed both before and after CE using different routes, the prior DBE was negative while the second DBE procedure guided by CE confirmed the lesion found at CE. These results suggest the use of CE to guide selection of DBE route.

### 3.6. Comparison of the Three Approaches in the Diagnosis of SBT

**Figure 1:**
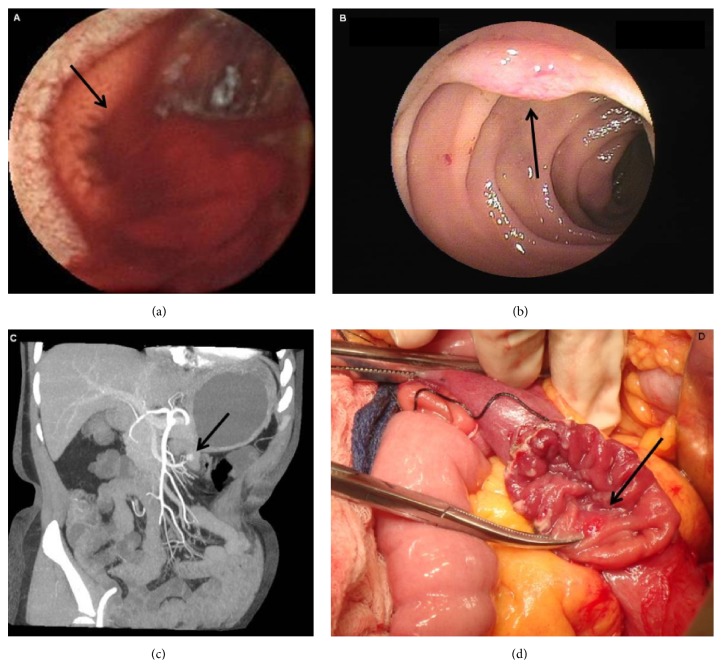
Images in a patient presenting with melena and diagnosed with hemangioma. (a) Image from capsule endoscopy showed active bleeding (arrow) in the proximal jejunum, without evidence of the source of bleeding. (b) Image from double-balloon enteroscopy indicated protruding lesion (arrow) with superficial vascular dilatation in the proximal jejunum. (c) Dual-phase computed tomographic enterography showed a lesion (arrow) in the jejunum with striking enhancement in the arterial phase. (d) Macroscopic appearance of the dissected segment of jejunum with the lesion (arrow).

**Figure 2 fig2:**
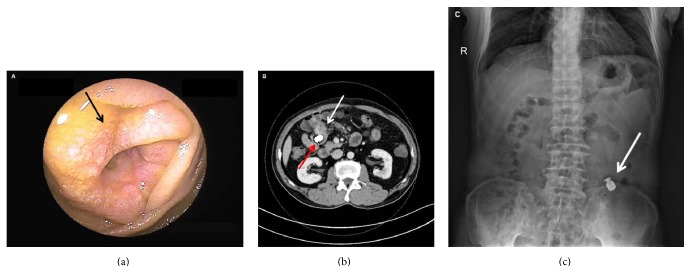
Images in a patient presenting with melena and diagnosed with adenocarcinoma. Capsule endoscopy was retained near the lesion, with no clear picture showing the lesion. (a) Image from double-balloon enteroscopy indicated a circumferential protruding lesion (arrow) with narrowed lumen in the proximal jejunum. (b) Dual-phase computed tomographic enterography showed a protruding lesion (white arrow) and retained capsule (red arrow) in the jejunum. (c) X-ray revealed the retained capsule (arrow).

**Figure 3:**
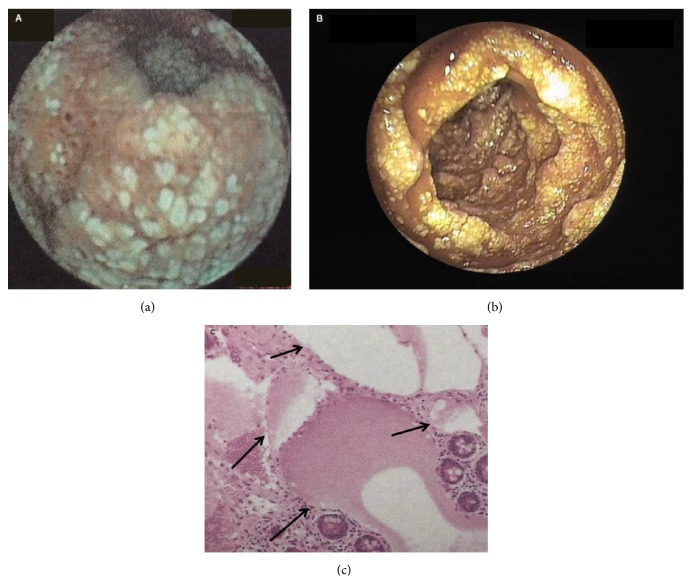
Images in a patient presenting with melena and diagnosed with lymphangioma. (a) Image from capsule endoscopy demonstrated circumferential white granular protruding lesions in the small bowel. (b) The same lesion was found in flakes in the proximal ileum during double-balloon enteroscopy. (c) Biopsy (hematoxylin and eosin stain) taken at surgery showed markedly dilated lymphatic channels (arrows) involving part of the bowel wall.

Small bowel tumors comprised the second leading cause of OGIB in this study. In the 27 patients diagnosed with SBT by combined approaches, using pathology as gold standard, gastrointestinal stromal tumor (GIST) was the most common type of tumor. In most cases (77.8%), tumor was located in the jejunum, and the remaining were found in the ileum (4 cases), duodenum (1 case), and both jejunum and ileum (1 case). CE detected tumors in 15/27 cases (sensitivity 55.6%, 95% confidence interval [CI] 35.3%–74.5%; specificity 100%, 95% CI 96.2%–100%), CTE was positive in 15/21 cases (sensitivity 71.4%, 95% CI 47.8%–88.7%; specificity 97.5%, 95% CI 91.2%–99.7%), and DBE identified tumors in 15/17 cases (sensitivity 88.2%, 95% CI 63.6%–98.5%; specificity 100%, 95% CI 88.1%–100%). The findings of CE, CTE, and DBE tests in those cases are compared in Table 5. Three representative cases are shown in Figures 1 [Fig fig2]–3. Twenty-five patients received all three examinations in this study, and SBT was diagnosed in 12 of them. CE and CTE each detected 6/12 tumors (sensitivity 50%; 95% CI 21.1%–78.9%), and DBE found 9/12 tumors (sensitivity 75%; 95% CI 42.8%–94.5%). There was no significant difference in tumor detection by matched pair comparisons of the three tests.

## 4. Discussion

According to literature review, OGIB accounts for the most common indication for both CE and DBE examinations (66.0% and 62.5%, resp.), with diagnostic yields of 60.5% and 68% for CE and DBE, respectively [2, 3]. The complimentary value of CE and DBE in the diagnosis of OGIB has been intensively studied in recent years. However, investigations on the value of combined CE, DBE, and CTE have rarely been reported.

A meta-analysis showed the pooled overall diagnostic yields of CE and DBE, and detection rates of vascular, inflammatory, and neoplastic lesions were similar in patients with OGIB [4]. In another pooled analysis, a significantly higher yield of CE was shown compared with DBE performed using single routes, but the yield of CE for OGIB was lower than DBE approaches combining both oral and anal routes [5]. There was a good degree of concordance between CE and DBE in the detection of vascular and inflammatory lesions, but not for tumors [6, 7]. In our study, the overall diagnostic yields were similar between CE and DBE. The detection rate of vascular lesions was higher in CE compared with DBE (*p* = 0.013), but the identification of neoplastic lesions was not significantly different. We reviewed those negative DBE cases and found that DBE was performed prior to CE in half of them. Using single-route DBE in most patients as the initial test without the guide of CE result might account for its low yield. This is also likely due to the selection bias of this study. In clinical practice, CE and DBE are not performed routinely in the same patient with OGIB, unless the initial test is nondiagnostic or needs further confirmation. Since we only included patients who underwent CE combined with CTE and/or DBE in this study, it is likely we selected those cases difficult to be identified with initial DBE alone. However, we do not view this as a major limitation, since our objective was to evaluate the integration of three imaging modalities in the diagnosis of OGIB.

Several studies were conducted to compare the diagnostic value of CE and CTE in OGIB patients. In one study, 123 patients underwent both CE and CTE with the indication of OGIB, and positive detection rate was higher in CE than CTE (57.7% versus 30.1%, *p* < 0.01) [8]. In another study involving 58 patients, CTE was more sensitive than CE in detecting the bleeding sources in the small bowel (*p* = 0.008), with evident advantages in depicting tumors [9]. Other studies showed improved diagnosis when CTE was used in patients with negative initial CE or CE used following initial nondiagnostic CTE [10, 11]. In our study, the overall diagnostic yield was significantly higher in CE compared with CTE (*p* < 0.001), mainly because CE showed marked advantage in identifying angiodysplasia (*p* < 0.001), while the sensitivity of detecting tumors was comparable between the two.

Angiodysplasia is reported to be the top diagnostic finding for OGIB on both CE and DBE. Pooled analysis showed that vascular lesions comprise 50% and 40% of CE and DBE findings in patients with OGIB, respectively [12]. In this study, the diagnostic yield of angiodysplasia was higher at CE (66.9%) and lower at DBE (17.4%), compared with the average level reported in literature. This is likely due to the mixed sequences of CE and DBE tests, incomplete intestinal scrutiny by DBE, and selection bias caused by the nature of this study. Vascular lesions only account for a small proportion of positive findings at CTE in OGIB patients, because of the limitation of CTE in identifying flat lesions. Preliminary reports suggested the introduction of a triphasic CTE technique using multiple phases improved detection of bleeding sources, but not in mucosal lesions such as angiodysplasia and ulcers [13, 14].

Small bowel tumors, both benign and malignant neoplasms counted, comprise the second major finding in this study, mainly because we selected patients who underwent CE plus CTE and/or DBE, and those with highly suspected neoplastic lesions found at CE need confirmation from other diagnostic methods. The sensitivity of CE was lower than that of CTE and DBE in our study. The miss rate of CE in tumor detection was reported to be 18.9% in a pooled analysis [15]. Pooled diagnostic yield of SBT is higher for DBE compared with CE (22% versus 9%) [12], but CE was suggested as the third test after negative bidirectional endoscopies for its ability to improve diagnostic yield of follow-up DBE [16]. In our study, CE directed the diagnosis of SBT made by DBE in 60% of the cases. In 4 of the patients, CE detected abnormal blood in the intestine, but due to its inability to perform flushing or suctioning, mucosal visualization was blurred by the active bleeding. However, the findings facilitated follow-up DBE in locating the tumors as the bleeding lesion. CTE is a valuable method in detecting small bowel masses as well, due to its advantage in depicting bowel wall and extraintestinal lesions. CTE showed higher sensitivity than CE in finding SBT among patients with OGIB and proved useful in the diagnosis of SBT missed by CE [9, 17].

In our study, Crohn's disease diagnosed at CE was based on criteria proposed in the previous literature [18], and diagnosis was made in 5 patients, one of which was confirmed by CTE. Suspected inflammatory bowel disease is a major indication for CTE, but the role of CTE is limited in early Crohn's disease, as the superficial mucosal lesions in the early stage are not visible at CTE. This might explain the cases of CD suggested at CE but not supported by CTE. However, CE might be false-positive in the diagnosis because of its low specificity, as CE was reported to detect mucosal breaks and erosions in over 10% of normal subjects [19].

We also identified diverticula in 6 patients of this study, five of which were found at DBE; among them, three cases were detected at CE. The remaining one was found by CTE alone. CE and CTE are not the conventional procedures to detect diverticula, but they provide useful information to direct follow-up tests for confirmation of the lesion. DBE is a reliable tool in the diagnosis of diverticula before surgery, and diverticula were reported as the fourth major finding (5%) at DBE in patients with OGIB [3]. In a study reviewing 74 patients with confirmed Meckel's diverticula, DBE showed a diagnostic yield of 86.5% before surgery and demonstrated superior sensitivity in comparison with CE (*p* < 0.001) [20].

Our study has several limitations. First, it was a retrospective comparative study, and the subjects investigated were patients who underwent CE plus CTE and/or DBE procedures; thus they were not a true representation of the population with OGIB. The study design likely resulted in selection bias of patients with small bowel diseases that were indicated for combination of several techniques for diagnosis. Second, among those patients who underwent DBE, not all patients received total balloon enteroscopy, which led to underestimated yield of DBE procedure as compared with CE. Third, the CE, CTE, and DBE procedures were not performed in a fixed sequence, and the order of CE and DBE tests could affect their diagnostic yields.

## 5. Conclusions

The diagnostic yields of CE and DBE were comparable in patients with OGIB in our study settings, which were significantly higher than the yield of CTE. CE proved to be superior in the detection of angiodysplasia. The three approaches showed comparable performances in the identification of small bowel tumors. DBE and CTE identified small bowel diseases undetected or undetermined by CE. Conversely, CE improved diagnosis in the cases with negative CTE and DBE, and positive findings at initial CE directed further diagnosis made by DBE. Combination of the three diagnostic platforms in a properly integrated manner based on individual patient conditions provides complementary value in the diagnosis of OGIB.

## Figures and Tables

**Table 1:** Characteristics and findings of the study group (*N* = 121).

Parameters	Value
Age (years) (mean ± SD)	51.1 ± 17.1
Sex (M/F)	60/61
Test order	
Tests before CE (*n*/*N*)^a^	CTE: 77/100; DBE: 9/46^b^
Tests after CE (*n*/*N*)^a^	CTE: 23/100; DBE: 38/46^b^
DBE route	Oral alone: 22; anal alone: 19; combined: 5
Findings *n* (%)	
Angiodysplasia	83 (68.6)
Small bowel tumor	27 (22.3)
Diverticulum	6 (5.0)
CD	5 (4.1)

CE: capsule endoscopy; CTE: computed tomographic enterography; DBE: double-balloon enteroscopy; CD: Crohn's disease.

^a^Value given as the proportion of CTE and DBE tests done before or after CE over all tests performed.

^b^One patient had two DBE procedures, one before CE and one after CE.

**Table 2:** Comparison of findings on CE versus DBE (*N* = 46).

Findings	CE (*n*)	DBE (*n*)	Confirmed cases (*n*)	Miss rate (%)	
				CE	DBE
Angiodysplasia	18	8	20	10	60
Tumor	11	15	18	38.9	16.7
Diverticulum	3	5	5	40	0
CD	2	0	2	—^c^	—

CE: capsule endoscopy; CTE: computed tomographic enterography; DBE: double-balloon enteroscopy; CD: Crohn's disease.

^c^Limited sample size.

**Table 3:** Comparison of findings on CE versus CTE (*N* = 100).

Findings	CE (*n*)	CTE (*n*)	Confirmed cases (*n*)	Miss rate (%)	
				CE	CTE
Angiodysplasia	73	8	74	1.4	89.2
Tumor	10	15	21	52.4	33.3
Diverticulum	0	1	1	—	—
CD	4	1	4	—	—

CE: capsule endoscopy; CTE: computed tomographic enterography; DBE: double-balloon enteroscopy; CD: Crohn's disease.

**Table 4:** Comparison of findings on CE versus CTE versus DBE (*N* = 25).

Findings	CE (*n*)	CTE (*n*)	DBE (*n*)	Confirmed cases (*n*)	Miss rate (%)		
					CE	CTE	DBE
Angiodysplasia	10	1	2	11	9.1	90.9	81.8
Tumor	6	6	9	12	50	50	25
Diverticulum	0	1	0	1	—	—	—
CD	1	0	0	1	—	—	—

CE: capsule endoscopy; CTE: computed tomographic enterography; DBE: double-balloon enteroscopy; CD: Crohn's disease.

**Table 5:** Small bowel tumors diagnosed in this study (*N* = 27).

Patient	Sex^d^	Age	Tumor type	CE	CTE	DBE	Test order^e^
1	F	57	GIST	Negative	Tumor	N/A	C-T
2	M	36	GIST	Active bleeding	Tumor	N/A	C-T
3	M	47	Lymphangioma	Tumor	Negative	Tumor	T-C-D (o)
4	F	56	GIST	Tumor	Tumor	N/A	C-T
5	F	62	GIST	Active bleeding	Tumor	Tumor	C-T-D (a)
6	M	49	Adenoma	Active bleeding	Negative	Tumor	T-C-D (o)
7	F	57	Hemangioma	Active bleeding	Tumor	Tumor	C-T-D (o)
8	F	55	GIST	Angiodysplasia	Tumor	N/A	C-T
9	F	44	Lymphangioma	Tumor	Negative	Tumor	T-C-D (o)
10	F	49	GIST	Tumor	Negative	Tumor	T-C-D (o)
11	M	64	Adenoma	Tumor	Negative	Tumor	T-C-D (o)
12	F	73	Lymphoma	Angiodysplasia	Tumor	N/A	C-T
13	F	74	GIST	Tumor	Tumor	N/A	T-C
14	M	51	Metastatic tumor	Tumor	Tumor	N/A	C-T
15	M	51	GIST	Angiodysplasia	Tumor	N/A	C-T
16	F	70	GIST	Negative	N/A	Tumor	C-D (a)
17	M	66	Adenocarcinoma	Negative	Tumor	Tumor	C-T-D (o)
18	M	51	GIST	Nonspecific enteritis	Negative	Tumor	T-C-D (a)
19	M	55	GIST	Tumor	N/A	Tumor	C-D (o)
20	F	43	Hemangioma	Tumor	Tumor	Negative	T-C-D (o)
21	M	54	Adenocarcinoma	Tumor	Tumor	Negative	T-C-D (o + a)
22	F	51	GIST	Tumor	N/A	Tumor	C-D (o)
23	F	33	GIST	Tumor	N/A	Tumor	C-D (o)
24	M	71	GIST	Angiodysplasia	Tumor	Angiodysplasia	C-T-D (a)
25	F	49	GIST	Tumor	N/A	Tumor	C-D (o)
26	F	60	Adenocarcinoma	Tumor	N/A	Tumor	C-D (o)
27	F	46	GIST	Tumor	Tumor	N/A	T-C

CE, CTE, and DBE were performed in 27, 21, and 17 of the 27 patients, respectively.

CE: capsule endoscopy; CTE: computed tomographic enterography; DBE: double-balloon enteroscopy; GIST: gastrointestinal stromal tumor; N/A: not applicable.

^d^Sex: M, male; F, female.

^e^Test order: C, CE; T, CTE; D, DBE; o, oral route; a, anal route.
